# Mother and Home Visitor Emotional Well-Being and Alignment on Goals for Home Visiting as Factors for Program Engagement

**DOI:** 10.1007/s10995-018-2535-9

**Published:** 2018-05-31

**Authors:** L. Burrell, S. Crowne, K. Ojo, R. Snead, K. O’Neill, F. Cluxton-Keller, A. Duggan

**Affiliations:** 1 Department of Population, Family and Reproductive Health, Johns Hopkins Bloomberg School of Public Health, 615 N. Wolfe Street, Baltimore, MD 21205 USA; 2 Dartmouth Geisel School of Medicine, 1 Rope Ferry Road, Hanover, NH 03755-1404 USA

**Keywords:** Home visiting, Maternal satisfaction, Engagement, Emotional well-being

## Abstract

*Objectives* Family engagement in home visiting (HV), as indicated by length of enrollment, is a major challenge as most families do not stay enrolled for the intended duration prescribed by HV models. This study examined maternal and visitor emotional well-being as factors for maternal satisfaction with the program in addressing reasons for enrolling in HV and program engagement and the role of their working alliance with the visitor as a mediator of this. *Methods* Longitudinal data were collected from 148 mothers and 54 visitors in 21 HV programs. Mothers completed surveys shortly after enrolling and 6 months later to assess attributes of the working alliance with their visitor. Visitors completed a survey to assess work-related well-being. HV program data were used to measure engagement. *Results* Mothers enrolled for multiple, diverse reasons, most often to promote child development and parenting (96%). Mothers’ satisfaction with program efforts to address reasons for enrollment was highest for parenting (79%) and lowest for jobs and education (30%). Results of the mediational path model indicated that ratings of the visitor on goal alignment were positively associated with engagement. Maternal emotional availability and visitor work-related emotional exhaustion were negatively associated with engagement. Exploratory analyses suggested that ratings of the visitor on goal alignment were a stronger predictor of engagement for mothers with low emotional availability compared to other mothers. *Conclusions for Practice* Visitor alignment with mothers on goals and responsiveness to reasons for enrolling appear to be effective in promoting engagement. Individualizing services to reflect maternal goals and emotional capacity may be important strategies to address engagement challenges.

## Significance

Family engagement continues to be an important issue facing home visiting field; studies find that most families do not receive the intended amount of services. Previous studies have documented attributes of visitors and families that are associated with HV engagement. This study extends previous investigations into attributes of mothers and visitors associated with HV engagement by considering mothers’ reasons for enrolling and their visitors’ alignment with and attention to these reasons.

## Introduction

Family engagement, most commonly defined as the length of enrollment or the frequency of home visits, has been a major challenge for home visiting (MIECHV TACC, 2015). Most evidence-based home visiting models intend to serve families for 2–3 years with intensive visit schedules and research suggests that engagement rates fall well below expectations. In fact, typically 40–50% of families leave within 1 year (Boller et al. 2014; Duggan et al. 2007). Because engagement remains a challenge, research to promote it is a national priority (Duggan et al. 2013).

Studies have documented attributes of visitors and families that are associated with engagement (Cho et al. 2017; Latimore et al. 2017; O’Brien et al. 2012). For example, Latimore et al. (2017) found that visitor morale was associated with receiving a high dose of services. Maternal age, race, and pregnancy status are commonly associated with engagement (Daro et al. 2003; McGuigan et al. 2003; O’Brien et al. 2012). Other maternal characteristics, including depression and relationship security, may influence engagement (Girvin et al. 2007; Sharp et al. 2003). Relationship security is the extent to which an adult provides and receives support in relationships, including one formed between a mother and visitor. McFarlane et al. (2010) found that mothers with anxious relationship styles were more likely to receive a high dose of service, suggesting that they may be particularly open to developing a close relationship with the visitor. Mikulincer and Nachshon (1991) found associations between relationship avoidance and a lack of self-disclosure, which may make it harder for a visitor to identify the mother’s needs.

Home visiting values a strong alliance between visitors and mothers to help mothers reach their goals. Mothers choose to enroll for many reasons, such as wanting information about job training, child development, and tangible assistance (Stevens et al. 2005; Tandon et al. 2008). Visitors respond to these reasons in varied ways. For instance, Tandon et al. (2008) found that visitors were most likely to meet the needs that were more proximal to program goals such as parenting information.

The nature of the mother’s working alliance with the visitor is predictive of family engagement. Studies have shown that mothers with better relationships with their visitors were more likely to have higher levels of program involvement and complete the program (Girvin et al. 2007; Korfmacher et al. 2007). Evidence suggests that this working alliance may be impacted by characteristics of the mother and the visitor. For example, mothers with both severe depression and discomfort with trusting others had lower ratings of trust in their visitor (Cluxton-Keller et al. 2014).

The Maternal, Infant, and Early Childhood Home Visiting (MIECHV) Program was established in 2010 to expand evidence-based home visiting and to carry out research to study its implementation and effectiveness.^1^ The current study aims to: (a) describe mothers’ reasons for enrolling, (b) examine the associations of maternal and visitor characteristics with mothers’ ratings of their working alliance with their visitors, (c) examine the association of attributes of the working alliance with program engagement, and (d) explore the role of the working alliance as a mediator of associations of maternal and visitor characteristics with program engagement.

## Methods

### Setting and Participants

With State and Federal MIECHV funding, the New Jersey Departments of Health and Children and Families are collaborating to build the state’s home visiting program (NJ-HV). The evaluation of NJ-HV incorporates mixed methods to inform strategies to improve engagement and outcomes by strengthening the implementation system. This paper uses data from a longitudinal study of a purposive sample of mothers newly enrolling in home visiting. The research was conducted in accord with prevailing ethical principles and approved by the Johns Hopkins School of Medicine and state Institutional Review Boards. Study participants provided written informed consent and were remunerated for data collection.

In 2012, NJ-HV funded 33 home visiting program sites statewide. Each site used one of three evidence-based models: Healthy Families America (HFA), Nurse-Family Partnership (NFP), and Parents as Teachers (PAT). Sites were eligible for this study if they had operated for at least 2 years^2^ and had at least three visitors who had completed a web-based survey. Visitors were eligible if they had completed core training and were able to visit families independently. In total, 95 visitors in 21 program sites were eligible for this study (27 visitors across 13 HFA sites, 27 visitors across 7 NFP sites, 3 visitors from 1 PAT site).

A maximum of three mothers per eligible visitor were recruited between June 2012 and March 2013. Researchers trained visitors to identify eligible mothers and introduce the study within the first three visits. A mother was eligible for the study if she: (a) was pregnant or had an infant less than 3 months old; (b) received services in English or Spanish; and (c) was assigned to a visitor who had not recruited three families.

During recruitment, 730 mothers enrolled in services. Of those enrolled, 536 were eligible for the study. Of the 536, researchers received contact information for 205. Primary reasons for not receiving information included mother declined to participate (47%), the study was not yet introduced to the mother, but she did not have at least three visits (e.g. family was discharged) (31%), and the study was not introduced to the mother during the first three visits (16%). Of the 205 mothers who agreed to be contacted by the research team, 177 (86%) consented and completed a baseline survey.

### Data Sources

#### Maternal and Visitor Surveys

The baseline maternal surveys included questions about demographic characteristics, psychosocial well-being (e.g. depression and relationship security), and maternal and family functioning. They completed follow-up surveys 6 months later or, if pregnant at baseline, when the child was 6 months. The follow-up surveys included questions from baseline, but also included measures of child development, parent–child interaction, and ratings of home visiting.

Visitors completed web-based surveys in early 2012. The survey captured demographic information, ratings of workplace culture, training, supervision, and psychosocial aspects of work (e.g. emotional exhaustion and morale).

#### Program Management Information Systems (MIS)

NJ HFA and PAT sites use a web-based, MIS developed by the State University of New York and NJ NFP sites use ETO provided by the NFP National Service Office. These systems enable sites to document service delivery.

### Measurement

#### Maternal Baseline Measures

##### Emotional Availability

Emotional availability includes depressive symptoms and relationship security as research has confirmed a link between them (Roberts et al. 1996; Strodl and Noller 2003). Each dimension of attachment has characteristics that can make one vulnerable to developing depression (Mikulincer and Shaver 2007). To assess depressive symptoms, we used the 12-item, *Center for Epidemiologic Studies Depression Scale—Short Form* (Ross et al. 1983). A total score was derived by summing items and scores ≥ 10 indicated moderate to severe symptoms. Relationship security was measured using the 29-item, *Attachment Style Questionnaire-Short Form* (Karantzas et al. 2010). An exploratory factor analysis identified items loading onto anxiety and avoidance factors. The relationship anxiety subscale included 12 items (α = 0.85) and the relationship avoidance subscale included eight items (α = 0.76). Items were summed to create relationship anxiety and avoidance scores and then dichotomized such that scores above the theoretical median were considered ‘high.’ Mothers were considered low on emotional availability if they scored ‘high’ on relationship avoidance and scored positive for depressive symptoms or high on relationship anxiety.

##### Reasons for Enrolling in Home Visiting

Mothers were given a list of 27 reasons for enrolling (e.g. “Want help finishing education”) and were asked whether each was applicable. These reasons were grouped into 10 broad domains. Mothers were counted as enrolling for each domain if they endorsed any of the items within it.

#### Maternal Follow-Up Measures

##### Rating of the Visitor on Goal Alignment

The 4-item, goals subscale from the *Working Alliance Inventory—Home Visiting Short Form* (Boller et al. 2014; Horvath and Greenberg 1994) was used to assess the mother’s rating of her visitor regarding goals for home visiting (e.g. “My visitor and I are working toward mutually agreed upon goals”). Items were summed to yield overall scores ranging from 4 to 28 with high scores indicating greater alignment.

##### Rating of Satisfaction with the Program’s Efforts to Address Reasons for Enrolling

For each endorsed reason, mothers were asked how satisfied they were with the program’s efforts to meet each need. A continuous score (range from 0 to 100) was created that represented the proportion of the reasons where the mother indicated being completely or very satisfied.

#### Visitor Measures

##### Perceptions of Organizational Climate

Three first-order climate subscales of the *Organization Social Context Measurement System* (OSC; Glisson et al. 2008) were used to measure individual-level perceptions of emotional exhaustion, personalization, and personal accomplishment. Raw scores were converted to standardized T-scores with a mean of 50 and standard deviation of 10. High scores on personalization and personal accomplishment are desirable and low scores are desirable for emotional exhaustion.

##### Morale

The OSC also includes an individual-level measure of morale. Raw scores were converted to standardized T-scores with a mean of 50 and standard deviation of 10. High scores are desirable.

#### Program Engagement

Engagement was defined as the total number of days from program enrollment to last completed home visit. For families still active, total number of days was calculated to the last home visit as of March 31, 2017.

### Analytic Plan

Of the 177 mothers completing a baseline survey, 148 completed the follow-up (76 HFA, 70 NFP, and 2 PAT). When possible, analyses included all 148 mothers to maximize study power to detect meaningful differences. Analyses including visitor characteristics were limited to the 100 mothers whose visitor had completed a survey. These 100 mothers were served by 54 unique home visitors (31 HF, 21 NFP, and 2 PAT). Mothers with a follow-up survey and mothers with visitor surveys did not differ from those without on baseline characteristics.

Simple linear regression was used for bivariate analyses. Path analysis was used to simultaneously test the direct effects of maternal and visitor characteristics on engagement and the mediating effects of ratings of the visitor and program satisfaction. Variables were considered for inclusion in the model if they were significant at the bivariate level (p < .10). To account for clustering of families within visitor, we used a full information maximum likelihood estimator with standard errors, parameter estimates, and a Chi square test statistic robust to nonnormality of the outcome variable and non-independence of observations (Graham 2009; Muthen and Muthen 1998–2017). Several measures were used to evaluate model fit, including Chi square, RMSEA, SRMR, CFI, and TLI. The path analysis was performed using Mplus v7.4.

## Results

Mothers were young and most enrolled prenatally (Table 1). They were diverse in terms of race. About a third had education beyond high school and 20% were working. A third of mothers had low emotional availability. Half of visitors had a bachelor’s degree or higher (Table 2). They had been in their positions an average of 4.7 years. Visitors reported high levels of morale, with the average score > 60.

**Table 1:** Baseline maternal characteristics (n = 148)

Maternal age in years (mean (SD))	22.7 (5.9)
Teen mother (< 19 years old)	36%
Enrolled prenatally	71%
Race/ethnicity	
Hispanic/Latina	24%
Black/African American	39%
White, Non-Hispanic	30%
Other	7%
Married/living with partner	28%
Highest grade completed	
< HS	28%
HS/GED	32%
> HS, no degree	27%
Degree past HS	12%
Currently working	20%
Receiving TANF/welfare	19%
Low emotional availability	32%

**Table 2:** Visitor characteristics (n = 54)

Age	
< 30	21%
30–39	23%
40–49	33%
50+	23%
Race	
Hispanic/Latina	33%
Black/African American	28%
White, Non-Hispanic	35%
Other	4%
Highest level completed in school	
HS/GED	14%
Some college, no degree	24%
Associate’s/vocational	12%
Bachelor’s or higher	51%
Years in position (mean (SD))	4.7 (3.9)
Organizational climate^a^ (mean (SD))	
Emotional exhaustion	50.7 (8.1)
Personalization	49.4 (8.7)
Personal accomplishment	57.8 (7.1)
Morale^a^ (mean (SD))	60.2 (7.6)

^a^Raw scores converted to standardized T-scores with mean of 50 and SD of 10

### Reasons for Enrolling and Satisfaction with the Program’s Efforts

Most mothers enrolled for many reasons; 82% endorsed reasons in four or more of the domains. Nearly all enrolled for child development and parenting and healthy pregnancy and child health (Table 3). Only a sixth enrolled for mental health. Reasons for enrolling varied by select characteristics, such as pre- or postnatal enrollment, ethnicity, and emotional availability. For example, compared to other mothers, mothers with low emotional availability enrolled for more reasons (6.6 vs. 5.7, p = .01) and were more likely to enroll for reasons related to education and employment (77 vs. 53%, p = .01) and other economic needs (58 vs. 40%, p = .04).

**Table 3:** Mothers’ reasons for enrolling in home visiting and satisfaction with program efforts (n = 148)

	% Mothers enrolling for this reason	% Satisfied with program efforts^a^
Child development and parenting	96	79
Healthy pregnancy and child health	94	63
Social support	77	57
Education and employment	61	30
Income and benefits	56	50
Primary care and insurance	56	47
Family planning	55	73
Other economic needs^b^	46	31
Other maternal health and wellbeing^c^	38	70
Mental health	16	56
Mothers’ ratings of the visitor and program (mean (SD))		
Maternal rating of visitor on goal alignment^d^		23.3 (4.1)
Overall satisfaction with program efforts^e^		71.3 (26.8)

^a^Completely or very satisfied with program efforts for each reason noted within the domain

^b^Includes independence from TANF and housing

^c^Includes healthy adult relationships, help with smoking, and help with alcohol

^d^Possible range 4–28

^e^Possible range 0–100

Overall, mothers rated their visitors favorably on goal alignment (Table 3). Scores ranged from 9 to 28, with an average of 23.3 (SD = 4.1). Most mothers reported being very or completely satisfied with the program’s efforts around child development and parenting (79%). They were least satisfied with efforts related to education and employment. On average, mothers were satisfied with program efforts on 71% of their reasons for enrolling.

### Bivariate Associations of Maternal and Visitor Characteristics with Ratings of the Visitor and Satisfaction with Program Efforts

Maternal age and visitor morale were positively associated with ratings of the visitor on goal alignment (Table 4). In contrast, low emotional availability and visitor emotional exhaustion were negatively associated with these ratings.

**Table 4:** Bivariate associations of maternal and visitor characteristics with mothers’ ratings of the visitor and satisfaction with program efforts

	Rating of visitor on goal alignment		Overall satisfaction with program efforts	
	B	p	B	p
Maternal characteristics				
Age	0.12	.04	− 0.12	.74
Hispanic	− 0.12	.87	− 1.92	.71
Enrolled prenatally	0.15	.84	13.44	< .01
Low emotional availability	− 1.97	< .01	− 7.74	.10
Visitor characteristics^a^				
Organizational climate				
Emotional exhaustion	− 0.09	.08	0.04	.92
Personalization	0.03	.50	− 0.21	.53
Personal accomplishment	0.07	.30	− 0.36	.38
Morale	0.12	< .01	− 0.11	.83

^a^Limited to mothers with a visitor who completed the web-based staff survey (n = 100). Models are clustered on visitor

Prenatally enrolled mothers were more likely to report satisfaction with program efforts to address their reasons for enrolling; mothers with low emotional availability had lower satisfaction scores.

### Factors for Program Engagement—Bivariate Associations

On average, mothers were enrolled for 616 days. Days enrolled increased as maternal age increased, Hispanic mothers were enrolled longer than non-Hispanic mothers and mothers with low emotional availability were enrolled fewer days than other mothers (Table 5). Visitor emotional exhaustion and morale were significantly related to engagement.

**Table 5:** Factors for program engagement, bivariate associations

Bivariate associations	Total days enrolled in home visiting program	
	B	p
Maternal characteristics		
Age	10.5	.07
Hispanic	133.0	.10
Enrolled prenatally	67.6	.37
Low emotional availability	− 174.2	.02
Visitor characteristics^a^		
Organizational climate		
Emotional exhaustion	− 13.4	.01
Personalization	4.6	.33
Personal accomplishment	-0.9	.89
Morale	10.1	.09
Maternal ratings of the visitor and program		
Maternal rating of visitor on goal alignment	24.1	< .01
Overall satisfaction with program efforts	3.4	.01

^a^Limited to mothers with a visitor who completed the web-based staff survey (n = 100). Models are clustered on visitor

Mothers’ ratings of the visitor on goal alignment and their satisfaction with the program’s effort to address reasons for enrolling were positively associated with engagement.

### Factors for Program Engagement—Mediational Path Model

**Fig. 1:**
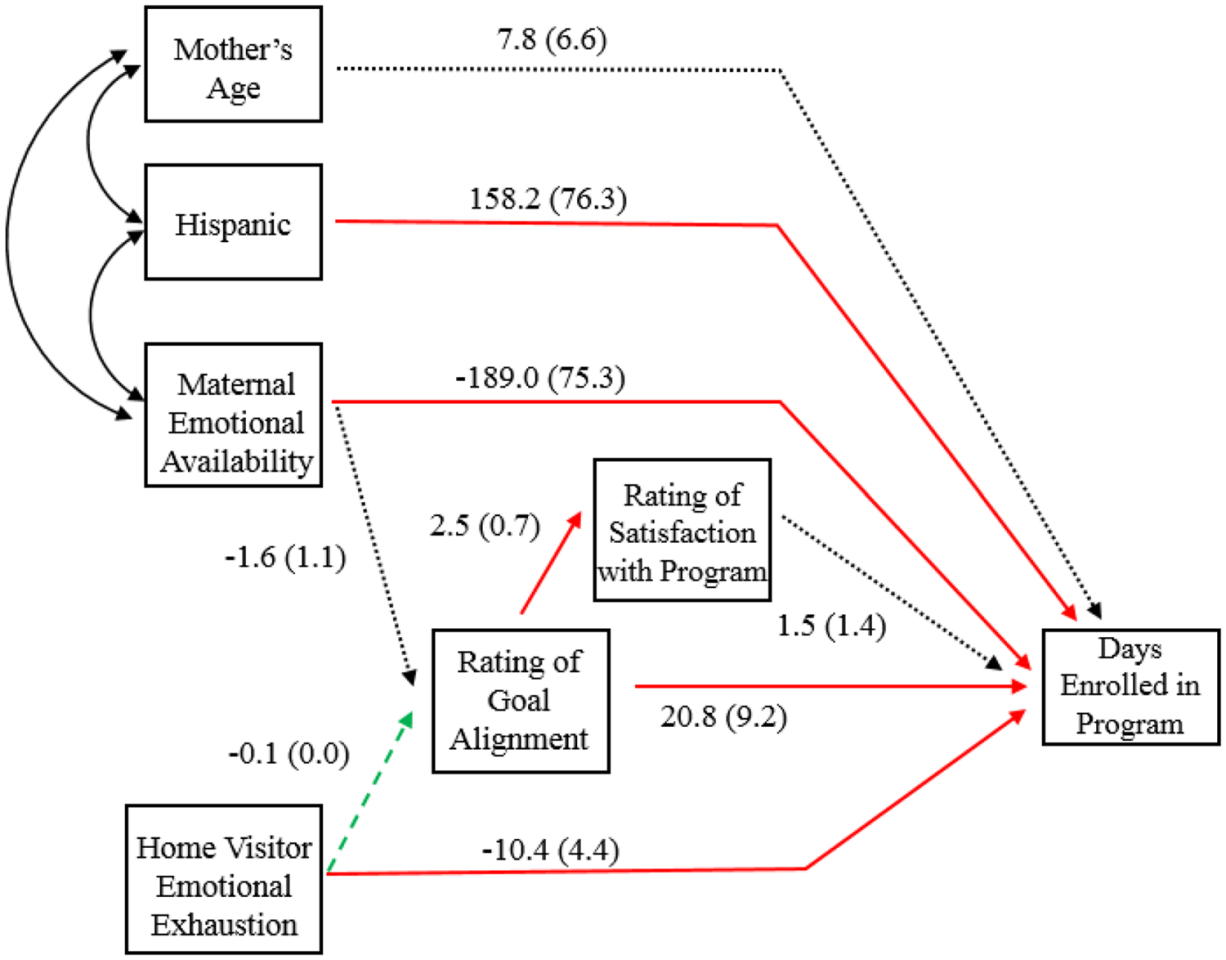
Direct effects of mother and visitor characteristics on days enrolled in the program. Red, solid arrows represent statistically significant direct effects at p < .05. Green, dashed arrows represent direct effects significant at p ≤ .10, and black, dotted arrows represent non-significant direct effects (p > .10). Curved arrows between maternal characteristics represent controlling for confounding effects of ethnicity and age in the model. Significance levels are not represented in curved arrows. Fit statistics: χ2 (9): 9.232, RMSEA = 0.016, SRMR = 0.054, CFI = 0.995, TLI = 0.991. (Color figure online)

In the path model, low maternal emotional availability and higher visitor emotional exhaustion were significantly associated with fewer days enrolled after controlling for age and ethnicity (Fig. 1). Ratings of the visitor on goal alignment were positively associated with number of days enrolled.

In this model, the total mediational effect of ratings of the visitor on goal alignment and program satisfaction on the relationship between emotional availability and length of enrollment were not statistically significant (*B* = − 40.03, p = .15). Likewise, ratings of the visitor and program did not significantly mediate the relationship between visitor emotional exhaustion and length of enrollment (*B* = − 2.08, p = .14). Fit statistics for this model all fell within the good to very good range.

To further explore these pathways, we repeated these analyses separately for mothers with low and adequate emotional availability. Visitor emotional exhaustion was significantly associated with fewer days enrolled, but only for mothers with adequate emotional availability (not shown; *B* = − 13.89, p = .01). Additionally, ratings of goal alignment were significantly associated with longer enrollment, but only for mothers with low emotional availability (not shown in figure; *B* = 32.83, p = .01).

## Discussion

This study investigated the associations among mother and visitor psychosocial characteristics, mothers’ ratings of the visitor on goal alignment, and satisfaction with program efforts to address their needs with length of enrollment. Mothers enrolled in home visiting for many reasons, were most satisfied with program efforts to address reasons related to parenting and child development and generally rated their visitors highly on goal alignment. Mothers who rated their visitor highly on goal alignment stayed in the program longer. Additionally, low emotional availability and visitor emotional exhaustion were both negatively associated with a family’s length of enrollment. Mothers with low emotional availability left home visiting about 6 months earlier than other mothers. Ratings of the visitor and program did not mediate the association of maternal and visitor characteristics on program engagement. Exploratory analyses suggested that ratings of the visitor on goal alignment were a stronger predictor of engagement for mothers with low emotional availability compared to mothers with adequate emotional availability.

These findings are consistent with other work, particularly Korfmacher et al. (2007), who found that maternal ratings of the relationship with her visitor predicted the amount of time spent in the program. The current study extends previous research by incorporating maternal and visitor characteristics and ratings of their relationship to help explain the effect on engagement.

Addressing maternal psychosocial risks is a challenge; studies have cited mental health concerns as one of the most common challenging situations facing visitors (Harden et al. 2010; Tandon et al. 2008). Although only 16% of mothers in this study enrolled for mental health reasons, one-third of mothers experienced low emotional availability, defined here as high levels of relationship avoidance combined with depressive symptoms and/or high levels of relationship anxiety. This is similar to rates found in other studies (Cluxton-Keller et al. 2014; McFarlane et al. 2013). These mothers were at a substantial risk of leaving the program prematurely and these findings suggest that this risk may be decreased when mothers and visitors are aligned on goals.

Visitor emotional exhaustion was also negatively associated with engagement. In the path model, emotional exhaustion was associated with a decrease in goal ratings. It is possible that visitors who are more emotionally exhausted are less responsive to addressing goals of mothers. And in fact, the exploratory results suggest that this is of particular concern when serving mothers with adequate emotional availability; when visitors are more exhausted, these mothers leave the program sooner. This would indicate that the work-related characteristics of visitors are important, particularly when serving mothers with adequate emotional availability.

We did not find that satisfaction with program efforts was a predictor of days enrolled in the path model. This is consistent with Korfmacher et al.’s (2007) finding that when both ratings of the helping relationship and satisfaction with the program were included in models together, ratings of the program were not as strong of a predictor of engagement as direct ratings of the visitor.

In this study, Hispanic mothers endorsed more reasons for enrolling, were more likely to enroll for reasons related to primary care, mental health, and maternal health and well-being, and stayed enrolled in the program longer than non-Hispanic mothers, which is consistent with other research (McGuigan et al. 2003; O’Brien et al. 2012). Given the transition to precision home visiting, the differentiation of what works, for whom, in what contexts to achieve specific outcomes, being led by HRSA, future work should examine how these pathways vary across race and ethnic groups.^3^

### Methodological Considerations

Study findings should be interpreted in light of methodological strengths and limitations. One strength is that the sample was drawn from a large number of local programs using three home visiting models. While the models vary slightly in who they target for services, all three models are similar in the intended length and intensity of services and in their primary outcomes for families (Michalopoulos et al. 2015). While the current study was not designed with the intention to test for differences across models, future work might consider whether models differ in how visitors work with families to set goals which align with their reasons for enrolling. Another strength is the use of path analysis. Since variables can be both independent and dependent, path analysis allows for the simultaneous estimation of all direct and mediating effects.

On the other hand, visitors played a role in recruiting the sample. This might have introduced bias. Also, sample size was small, particularly in analyses including visitor data, limiting power to detect meaningful associations. A post-hoc power analysis using a Monte Carlo simulation was carried out on the mediational path model and results indicated a lack of statistical power which could explain the non-significant, mediational effects.

A final limitation relates to measurement of family engagement. Duration of enrollment is easily measured, but is a simplistic indicator of true engagement. Future research should extend its focus to include measures of parents’ actual participation in visits as indicated by shared decision-making, observational measures of how well the visitor communicates with mothers to build partnership toward reaching goals, and how visitors tailor the content of visits to align with mothers’ goals.

### Implications

Family engagement in home visiting is a critical issue for the field. Visitor alignment with mothers on goals and responsiveness to reasons for enrolling appear to be effective in promoting engagement, especially for some mothers. Programs should formally identify mothers’ reasons for enrolling early on so that family goal plans, a strategy to build a strong working alliance, reflect the goals that are important to the family from the start. Implementation systems should be strengthened to support visitors in developing and using goal plans and with funding through a HRSA Innovation Award, New Jersey and Maryland are partnering to design and test elements of a goal plan strategy (GPS) implementation system.^4^ The purpose is to promote engagement by improving visitors’ competence to respond to a family’s reasons for enrolling, by making it easier for visitors to do this, and by reinforcing them in doing so.

Services should be individualized not only to a mother’s reasons for enrolling but also to her emotional availability. Most programs already screen for depression at enrollment or at a time relative to the child’s birth (Michalopoulos et al. 2015). Based on the current study, as well as our broader research, we feel it is important that programs consider relationship security in tailoring services. Work is needed to design and test strategies for this. Visitors are in a unique position not only to use formal screening tools but also to recognize and respond to mothers’ cues regarding their emotional well-being—both depressive symptoms and relationship security. If home visiting enrolls mothers with low emotional availability, we need to adjust our expectations of their engagement, our visitors’ strategies to personalize communication strategies to promote their engagement, or both. The high prevalence of low emotional availability underscores the importance of precision home visiting to improve on our current service models.

## Footnotes

1 For more information, see https://mchb.hrsa.gov/maternal-child-health-initiatives/home-visiting-overview.

2 For HFA sites, the site also had to have completed the national model’s site accreditation process.

3 For more information, see http://www.hvresearch.org.

4 HRSA Award # UH4MC307460100.
