# Correction to: Mother and Home Visitor Emotional Well-Being and Alignment on Goals for Home Visiting as Factors for Program Engagement

**DOI:** 10.1007/s10995-018-2621-z

**Published:** 2018-08-22

**Authors:** L. Burrell, S. Crowne, K. Ojo, R. Snead, K. O’Neill, F. Cluxton-Keller, A. Duggan

**Affiliations:** 1 Department of Population, Family and Reproductive Health, Johns Hopkins Bloomberg School of Public Health, 615 N. Wolfe Street, Baltimore, MD 21205 USA; 2 Dartmouth Geisel School of Medicine, 1 Rope Ferry Road, Hanover, NH 03755-1404 USA

## Correction to: Maternal and Child Health Journal 10.1007/s10995-018-2535-9

The article “Mother and Home Visitor Emotional Well-Being and Alignment on Goals for Home Visiting as Factors for Program Engagement”, written by L. Burrell, S. Crowne, K. Ojo, R. Snead, K. O’Neill, F. Cluxton‑Keller and A. Duggan, was originally published electronically on the publisher’s internet portal (currently SpringerLink) on 31 May 2018 without open access. With the author(s)’ decision to opt for Open Choice the copyright of the article changed on 5 July 2018 to © The Author(s) 2018 and the article is forthwith distributed under the terms of the Creative Commons Attribution 4.0 International License (http://creativecommons.org/licenses/by/4.0/), which permits use, duplication, adaptation, distribution and reproduction in any medium or format, as long as you give appropriate credit to the original author(s) and the source, provide a link to the Creative Commons license and indicate if changes were made.

The original article has been corrected.

